# Longitudinal Shedding Patterns and Characterization of Antibiotic Resistant *E. coli* in Pastured Goats Using a Cohort Study

**DOI:** 10.3390/antibiotics8030136

**Published:** 2019-09-02

**Authors:** Eunice Ndegwa, Hanin Almehmadi, Kim Chyer, Paul Kaseloo, Ankrah A. Ako

**Affiliations:** 1 Agricultural Research Station, Virginia State University, Petersburg, VA 23806, USA; 2 Department of Biology, Virginia State University, Petersburg, VA 23806, USA; 3 Department of Agriculture, Virginia State University, Petersburg, VA 23806, USA

**Keywords:** antibiotic resistance, goats, goat kids, resistant genes, virulence genes, phylogenetic grouping

## Abstract

There is a scarcity of information on antibiotic resistance in goats. To understand shedding of resistant *Escherichia coli* in pastured goats, we collected fecal samples from a mixed age cohort over a one-year period. No antibiotic had been used on the study animals one year prior to and during the study period. Resistant isolates were detected in all age groups and prevalence in goat kids was significantly higher than adults; 43–48% vs. 8–25% respectively. The proportion of resistant isolates was higher when animals were congregated near handling facility than on pasture. Most isolates were resistant to tetracycline (51%) and streptomycin (30%), but also to antibiotics that had never been used on the farm; ampicillin (19%). *TetB, bla_-TEM_*, (*aadA* and *strpA/strpB*) genes were detected in 70%, 43%, (44% and 24%) of tetracycline, ampicillin, and streptomycin resistant isolates respectively. Resistant isolates also harbored virulent genes and some belonged to D and B2 phylogenetic groups. Thus, pastured goats, despite minimal exposure to antibiotics, are reservoirs of resistant *E. coli* that may contaminate the environment and food chain and spread resistant genes to pathogenic bacteria and some that are potential animal and human pathogens. Environmental sources may play a role in acquisition of resistant bacteria in pastured goats.

## 1. Introduction

The emergence of antibiotic resistant pathogens and commensal bacteria is a phenomenon important to both human and animal health globally [1]. While the origins and reservoirs of antimicrobial resistance in the environment is a complex topic which continues to be a subject of intensive research [2], extensive use of antibiotics in animal agriculture is considered one of the major causes of emergence and spread of antibiotic resistance organism in the environment [3]. Recent studies, however, have reported antibiotic resistant isolates from animals who have never been exposed to antibiotic use [4] and in humans living in remote parts far away from any agricultural or close human activities [5]. These findings indicate that other factors may have a role in acquisition and dissemination of antimicrobial resistance genes in the environment [6].

Many studies on antimicrobial resistance of commensals and clinical isolates have evaluated resistance in food animals including poultry, swine, and cattle [7,8,9,10,11,12,13,14,15,16,17,18,19,20]. Many of these studies report a high prevalence of antimicrobial resistance in both commensal bacteria and pathogenic bacteria to a variety of clinically relevant antibiotics [21]. On the other hand, little has been published on status of antimicrobial resistance in small ruminants and little surveillance on the status of antimicrobial resistance in these species is reported globally [22]. While pastured small ruminants are generally reared under system of low antibiotic use, these animals interact freely with the environment under grazing systems. Furthermore, antibiotic resistant *Escherichia coli (E. coli)* have been detected in wild animals that have no known prior exposure to antibiotics [2,4]. Thus, it is potentially possible pastured small ruminants may acquire or spread antibiotic resistant bacteria and genes to the broader environment or ultimately get to the public and food chain through many pathways [5,23,24,25,26,27]. Due to the abundance in the animal gut, ease of acquisition of antimicrobial resistance genes, and ease of cultivation in the laboratory, *E. coli* is often used as a sentinel to understand status and for surveillance of antimicrobial resistance [28]. Furthermore, small ruminant’s gastrointestinal tract is host to *E. coli* strains that are of public health significance, including those that belong to the O157, O26, and O145 serotypes [28,29,30]. The characteristics of gut *E. coli* strains in goats has not been fully characterized, despite their importance as a meat and milk source globally. In this study, we evaluated the temporal shedding patterns of antibiotic resistant *E. coli* in a cohort of young goat kids, nursing does, and other adult goats in the cohort over a one-year period. We further characterized the resistant isolates genes responsible for the antimicrobial resistance phenotypes, virulence genes, and phylogenetic grouping of the various resistant isolates to further understand the public health significance of the isolates.

## 2. Materials and Methods 

### 2.1. Study Site Description and Livestock Management

The study cohort was a herd of Spanish and Myotonic goats which are part of Virginia State University (VSU), USA, research flock that are maintained on forty-five acres of land divided into fenced grazing paddocks and surrounded by wooded area. Animals are rotated between pasture lots year-round. A housing facility is available on site with small paddocks where animals routinely congregate in winter and for group procedures including hoof trimming, foot dips and during the weaning period. While grazing at the facility, animals are supplemented with baled hay. The breeding animals are routinely all bred in October/November and kidded on pasture around April/early May. During the experimental period, the animals were supplemented with hay as needed and a balanced corn-based concentrate. The animals are monitored daily during supplemental feeding for signs of ill health by the herd manager who communicated any concerns to the primary author and on all sampling days by the primary author. Interaction of the research animals and farm workers occur during supplemental feeding and during routine hoof trimming and deworming procedures. No antibiotics had been used on the participating animals at least one year before the initiation of the study and on both adults and kids in the study during the study period. Animals were cared for according to an approved Virginia State University Institutional Animal Care and Use Protocol (VSU 2017-02).

### 2.2. Study Groups and Sampling Protocol

Beginning May 2017, a cohort of 25 kids and their dams (15) were recruited in the study to understand and compare patterns of shedding of antibiotic resistant *E. coli* between nursing goat kids and their respective dams. The cohort was part of a larger flock and were all maintained on pastures and managed the same way throughout the study period. Animals in the initial cohort were first sampled at three weeks of age and thereafter monthly until weaning at about 3 months. In total, this cohort was sampled 4 times up to the weaning day. At 7 days post weaning, only kids in the initial cohort were sampled again to further understand shedding patterns around weaning time. To increase the sample size during subsequent samplings, the initial cohort of 25 kids and other goat kids born at the same period with the initial cohort and shared the same ecosystem and management were recruited into the study group. This latter group was sampled at six months and at one year of age.

For detection of shedding patterns based on location at the farm, 49 adult goats in the same flock under the same management were also included in the study. This group included animals sampled near the facility in March 2017 and on pasture in October 2017.

### 2.3. Fecal Sample Collection

At each sampling, individual fecal samples were collected from the rectum of kids and adults and transported in ice to laboratory for microbial isolation. The samples were processed the same day.100mg of fecal samples was mixed with 1ml of phosphate buffered saline and vortexed until the fecal pellet was well mixed with the saline. Samples were centrifuged at 700 rpm for three (3) minutes to sediment the fecal material and dilutions of supernatant were prepared and plated on MacConkey agar and incubated at 37 °C overnight. Two *E. coli* like colonies were picked from the plates with well separated colonies and transferred to Luria broth. Further transfer of isolates to Eosin Methylene Blue (EMB) agar was done for confirmation of *E. coli*. Colonies with metallic sheen growth on EMB media were assumed to be *E. coli*. Further confirmation utilized PCR amplification of 16S ribosomal gene amplification and visualization in agarose gel under UV light. Isolates were stored in 20% glycerol at −20 °C until processing for antimicrobial resistance.

### 2.4. Antimicrobial Susceptibility Screening

The standard Kirby–Bauer agar disk diffusion method was used to screen for antimicrobial resistance following recommendations by the Clinical and Laboratory Standards Institute (2015) recommendations. A total of 12 antimicrobial discs were used based on importance in animal and human health and included: ampicillin, gentamicin, amoxicillin-clavulanic acid, streptomycin, tetracycline, tobramycin, amikacin, trimethoprim-sulphamethoxazole, nalidixic acid, meropenem, chloramphenicol, and ciprofloxacin. One confirmed *E. coli* isolate per animal was screened unless isolates from the same animal displayed different colony morphology on EMB (mucoid vs non mucoid) in which case both were screened. *E. coli* isolates were transferred to Muller Hinton broth and incubated at 37 °C to 0.5 McFarand standard turbidity. 100 µL of the broth was subsequently spread onto Muller Hinton agar 150mm plates. The twelve (12) antibiotic discs were placed using a Thermo Scientific™ Remel™ Antimicrobial Susceptibility 12-place 150 mm Disk Dispenser, incubated overnight, and zones of inhibition measured for each of the tested antibiotic. American Type Culture Collection (ATCC) *E. coli* 25922 was used as the quality control reference strain in all the screening tests. For each individual antibiotic tested, antimicrobial susceptibility description (“resistant”, “intermediate”, and “susceptible”) was based on the criteria outlined in the CLSI manual 2015 for *Enterobacteriaceae*. Isolates that were found to be resistant to any of the tested antimicrobials were further processed for determination of the commonly detected antimicrobial resistance genes/mutations, virulence genes, and also for phylogenetic grouping.

### 2.5. Characterization of Antibiotic Resistance Genes

Overnight Luria broth culture of each resistant isolate was used for DNA extraction following a simple boiling method previously described [31] with a few modifications. In brief, 2 mL of overnight Luria broth containing the isolates was centrifuged at 14,800 rpm (full speed) for 3 minutes at room temperature. The supernatant was poured out and the pellet further re-suspended in one milliliter (1 mL) of molecular grade water by vortexing. The bacteria suspension was further centrifuged at full speed for 3 minutes. The supernatant was poured out and the pellet re-suspended by vortexing in 200–500 µL molecular grade water. The suspension was boiled for 20 minutes at 100 °C using a table top heating block to lyse the bacteria. The suspension was centrifuged again at full speed for 4 minutes to pellet the bacteria lysate and 150–300 µL of the supernatant containing the DNA transferred to a new tube. DNA concentration was measured using a Nanodrop 2000c and samples stored at −20 °C until further processing. The presence of genes encoding *TEM*, *OXA*, and *SHV* β-lactamases was studied by PCR in all the ampicillin-and amoxicillin /clavulanic resistant isolates. The following genes were also studied by PCR: *tet*(A), *tet*(B),*tet*(C), *tet*(D), and *tet*(E) (in tetracycline resistant isolates), *aadA* and *strA/B* (in streptomycin and tobramycin resistant isolates), *aac(3)-IV* (in gentamicin, tobramycin and amikacin-resistant isolates), *cmlA* (in chloramphenicol-resistant isolates), and *sul1*, *sul2,* and *sul3* (in trimethoprim–sulfamethoxazole-resistant isolates). The quinolone resistance-determining region (QRDR) of the *gyrA* gene was amplified in nalidixic acid resistant isolates [32] followed by sequencing using the same set of primers used for the PCR reactions. Sequences obtained were compared with those previously reported for *gyrA* (GenBank). The gene specific primers, their expected fragment sizes, and source references are listed in Table A1 (Appendix A) and were purchased from Thermofisher (Waltham, MA, USA). The gene products were visualized in ethidium bromide stained 1.5% agarose gel using UV light. A subset of PCR amplified fragment of the resistant genes were purified directly from the PCR reaction or after gel electrophoresis. The fragments were sequenced at MGH CCIB DNA Core (Cambridge, MA) and the resulting DNA sequence data were compared with corresponding genes in the GenBank database using the basic local alignment search tool (BLAST) of the National Center for Biotechnology Information web site (http://www.ncbi.nlm.nih.gov) to confirm identity of antimicrobial resistance genes.

### 2.6. Detection of E. coli Virulence Genes and Phylogenetic Grouping

Resistant isolates were screened for four virulence genes commonly found in pathogenic *E. coli*; shiga toxins 1 and 2 (*stx1* and *stx2*), hemolysin (*hly*), and also intimin (*eae*) by a polymerase chain reaction using gene specific primers listed in Table A1. The phylogenetic grouping of the isolates was also determined using primers targeting the *chuA*, *yjaA,* or *tspE4.C2* genes as previously described [33].

### 2.7. Statistical Analysis

Proportions of resistant isolates were calculated for all the groups sampled. Differences in proportion of resistant isolates among sampling groups were tested using the MedCalc N-1 Chi-squared Comparison of proportions calculator 2018 [34]. A *P* value of <0.05 was considered significant for all comparisons. Results of the statistical analysis are shown in Table A2 (Appendix B).

## 3. Results

### 3.1. Antimicrobial Resistance Proportions and Resistance Phenotypes

A total of 408 isolates that included (196) from both nursing does and kids up to weaning day, twenty-two (22) from kids one week after weaning, fifty-four (54) from goat kids at six months, forty-three (43) from goat kids at one year old, and ninety-three (93) from other goats in the flock were screened for antimicrobial resistance. We detected antibiotic resistant *E. coli* isolates in all age groups sampled in the absence of use of antibiotics in the study animals during the previous one year prior to initiation of the study and during the study period. In total, 136 isolates (33%) were resistant to one or more antibiotic, while 272 isolates (67%) were sensitive to all tested antibiotics. Most of the isolates 105 (77%) showed resistance to a single antibiotic, while 27 (20%) showed resistance to two antibiotics, and three (2.2%) isolates to three antibiotic and only one isolate with resistance to four antibiotics (Table 1). 

The highest resistance was reported for tetracycline (51%), followed by streptomycin (30%), ampicillin (19%), nalidixic acid (9%), amoxicillin/clavulanate (5%), and Chloramphenicol (5%), while for all the other antibiotics, resistance was less than 5% (Table 2).

In the group of isolates showing resistance to two or more antibiotics (31), most showed a combination of tetracycline and streptomycin (12) (39%), followed by ampicillin/amoxicillin clavulanate (3) (10%), while other combinations were less than 10% (Table 1). Overall, isolates with resistance to two or more antibiotics tended to be detected in older goats (6 months and older) more frequently than in younger goats (less than 6 months) (Table 3).

### 3.2. Proportions of Resistant E. coli Isolates and Phenotypes Among Age Groups and at Different Farm Location

**Figure 1:**
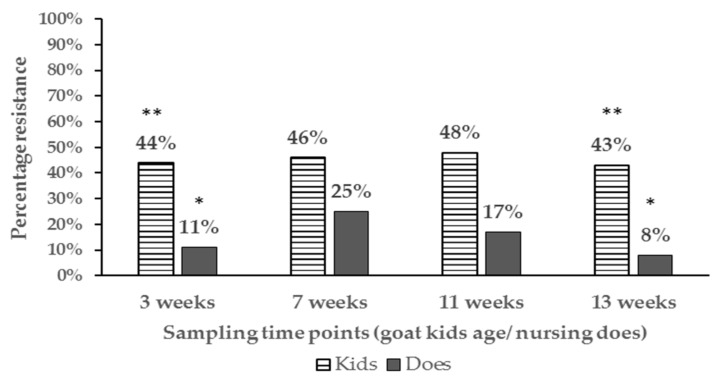
Bar chart illustrating percentage of resistant *E. coli* isolates in pastured goat kids and the respective nursing does up to weaning. Proportions at the same sampling points with different number of asterisks ** vs. * are significantly different (*P* < 0.05).

The proportion of antibiotic resistant *E. coli* isolates and resistance phenotypes detected differed among age groups. Resistant *E. coli* isolates in nursing goat kids ranged from 43% to 48% while that detected in the nursing does ranged from 8−25% up to 13 weeks of age (Figure 1).

**Figure 2:**
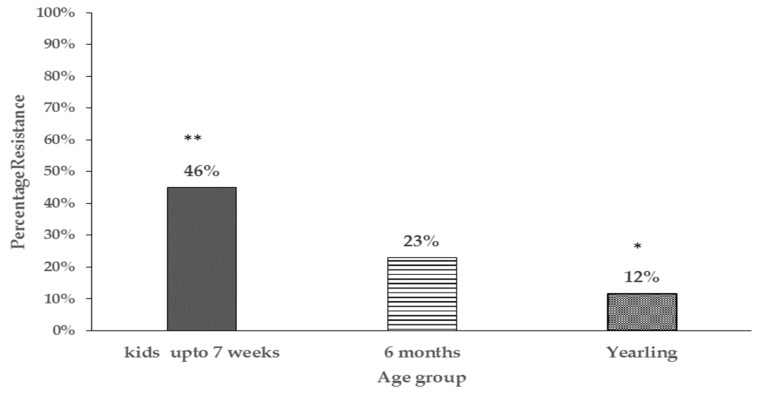
Bar chart comparing percentage of resistant *E. coli* isolates in different age groups of pastured goat kids (only goat kids sampled at pasture are represented). Proportions with different number of asterisks ** vs. * are significantly different (*P* < 0.05).

In general, the proportion of antibiotic resistant *E. coli* tended to be higher in goat kids compared to the does irrespective of location at the farm and significant differences (*P* < 0.05) were detected at 3 weeks and 13 weeks of age (Appendix A for table of statistical analysis results). Additionally, the percentage of resistant isolates in goat kids decreased with age, with significant differences being detected between goat kids at 3 and 7 weeks compared to one year of age (46% vs. 12%) respectively (Figure 2).

**Figure 3:**
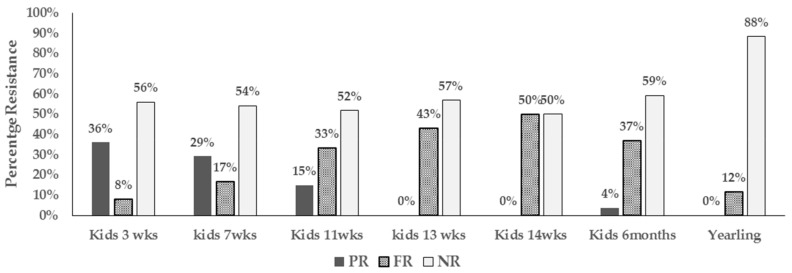
Bar chart illustrating types of resistant (intermediate vs full resistance) in *E. coli* from pastured goat kids from 3 weeks of age to one year (PR-Intermediate resistance, FR-Full resistance, and NR-non-resistance).

We also observed unique patterns in resistance phenotype detected in *E. coli* over the growing period. We noted the resistance detected in young goat kids at 3 weeks of age was mostly of intermediate type (PR). Isolates with intermediate resistance however decreased over time and by the time the kids were 13 weeks and beyond, resistance detected tended to be full resistance (FR) (Figure 3). 

This study also found that the resistance phenotype in nursing goat kids was unrelated to that detected in isolates from the respective nursing does despite the close interaction and shared ecosystem throughout the pre-weaning period. Resistance to streptomycin was the most prevalent during the first 14 weeks of age and was equally detected in both kids and does. However as mentioned above, resistance detected in young goat kids was mostly intermediate while that detected in nursing does was full resistance. Repeated fecal samplings also did not reveal persistence colonization by resistant isolates in any of the sampled animals over the study period. Instead, different resistance phenotype was detected in subsequent samplings and sometimes no resistant isolates was recovered at all in the same animal.

The proportion of *E. coli* isolates with antibiotic resistance phenotypes and also the type of resistance phenotype differed depending on the location of the animals (pasture vs paddocks around housing facility). A significantly higher percentage (*P* < 0.05) of antibiotic resistant isolates was detected in animals sampled while grazing at the paddocks surrounding the housing facility compared to animals sampled at pasture (Table 4). Additionally, tetracycline resistance was the predominant phenotype (31%) detected in isolates from animals at the facility compared to those located at pasture (2.5%). On the other hand, a significantly higher percentage of ampicillin resistant *E. coli* was detected in isolates from animals at pasture (11%) compared to those grazing around the housing facility (2%). The percentage of isolates resistant to streptomycin, nalidixic acid, chloramphenicol amoxicillin/clavulanate, and tobramycin did not differ significantly between *E. coli* isolates from animals at pasture or near the facility.

### 3.3. Antibiotic Resistance Mechanisms

To elucidate on possible mechanisms of antibiotic resistance in the *E. coli* from the goats in the study, all resistant isolates were screened for commonly encountered resistant genes for each antibiotic. The resistant genes detected in resistant isolates are shown in Table 5. Among 70 tetracycline resistant isolates, only *tetB* gene was detected in 69 isolates (97%) and no gene was detected in the other resistant isolate. In all ampicillin resistant isolates (28) the β- lactamases genes; *bla_TEM_*, *bla_OXA_*, and *bla_SHV_* were screened. The *bla_TEM_* gene was detected in twelve of the isolates (43%), while no gene was detected in the remaining isolates. One isolate harbored both the *bla_TEM_* and the *bla_SHV_* gene. The aminoglycoside modifying adenyltransferase enzyme gene *aadA* was detected in 18 of the 41 (44%) streptomycin resistant isolates. The *strpA/strpB* gene was also detected in ten (24%) of the streptomycin resistant isolates either alone or in combination with the *aadA* gene. Five streptomycin resistant isolates had both the *aadA* and *strpA/B* genes detected. The one isolate with resistance to sulfamethoxazole-trimethoprim harbored the s*ul2* gene while one isolate out of two with tobramycin resistance had the aminoglycoside acetyltransferase enzyme gene *aac3* (iv) detected. In the four isolates with amikacin resistance and seven with chloramphenicol resistance, no antimicrobial resistance gene was detected using the primers used in this study. Mechanism of resistance to nalidixic acid was evaluated by sequencing of the amplified fragment of *E. coli* gyrase gene. All resistant isolates showed a substitution mutation of both the Serine 83 leucine and asparagine 87 aspartate amino acids.

### 3.4. Virulence Genes and Phylogenetic Grouping of Resistant Isolates

**Figure 4:**
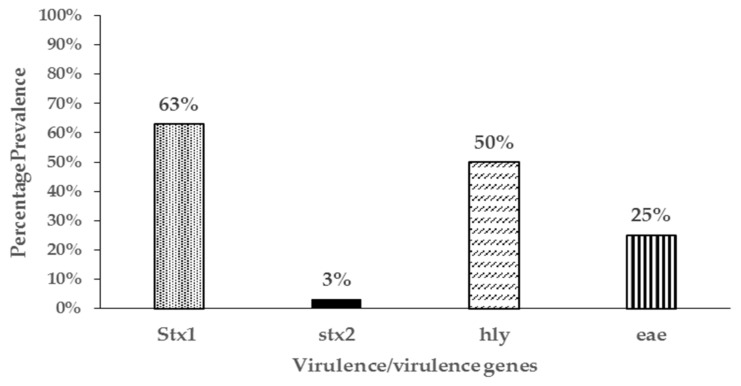
Prevalence of virulence genes in 104 antibiotic resistant *E. coli* isolates from pastured meat goats.

**Figure 5:**
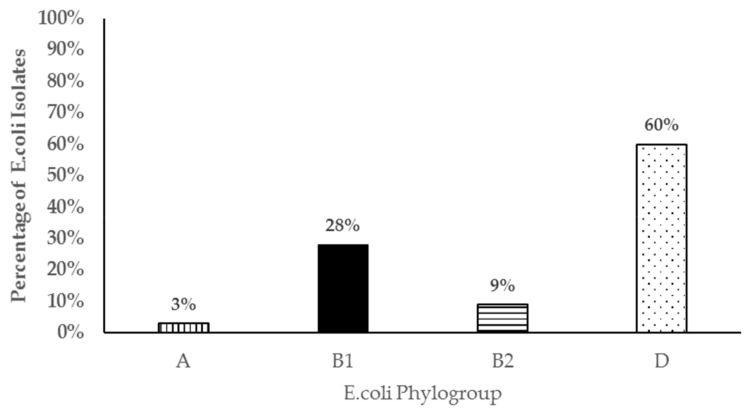
Bar chart representing phylogenetic groups of 104 antibiotic resistant *E. coli* isolates from pastured meat goats.

Four genes commonly associated with *E. coli* virulence in both animals and human were detected in resistant isolates in varying proportions. In one hundred and four (104) resistant isolates screened, shiga toxin 1(*stx1*), shiga toxin 2 (*stx2*), intimin (*eae*), and hemolysin (*hly*) genes were detected either individually or in combination in 84 (81%) of the resistant isolates (Figure 4). The most common virulence genes detected in the resistant isolates was for shiga toxin 1 (63%), followed by hemolysin (50%), and finally intimin (25%). Shiga toxin 2 (*stx2*) gene was only detected in two isolates. Resistant *E. coli* isolates were assigned to the four different phylogenetic groups based on the presence of the *chuA*, *yjaA* or *tspE4.C2* genes in different combinations. Sixty percent of the isolates (60%) belonged to the D group, 28% belonged to B1, 9% to B2, and 3% to the A phylogenetic groups (Figure 5). 

## 4. Discussion 

Surveillance of antibiotic resistance in food animals continues to be of importance worldwide due to the risk of spread of antibiotic resistance determinants to human pathogens, both zoonotic and non-zoonotic. Understanding the status of antibiotic resistance in animals is also important in veterinary medicine for disease control. Little is known on status or patterns of antibiotic resistance in small ruminants in North America, including the US [22], and little surveillance on the status of antimicrobial resistance in these species is reported globally.

Interestingly in this study, resistant *E. coli* isolates were detected in young goat kids on pasture as early as three weeks of age in the absence of use of any antibiotics in the goat kid cohort or the nursing does for the previous one year. While this was a unique finding not previously reported in goats, other studies have reported early colonization by resistant *E. coli* in young food animals of other species even before exposure to specific antibiotic or shortly after administration [35,36]. Furthermore, antibiotic resistant isolates were also reported in pristine ecosystems that have not been exposed to antibiotics before [2,36], which suggests that multiple factors may be responsible for antimicrobial resistance acquisition and spread in the environment. The role of horizontal transfer of resistant elements from environmental reservoirs in the soil or water or spread by wild animals has been thought to play a role [2] in some ecosystems, which may be the case in the current study, since the animals had not been treated with any antibiotics. In other studies, factors not associated with direct antimicrobial use, including geographic location, housing, animal age, and purpose of production, were also found to have an effect on the prevalence of *E. coli* resistant to antibiotics [7,9]. In this study, higher proportions of resistant *E. coli* isolates were detected in young goat kids than in nursing does in the same environment throughout the pre-weaning period. Prevalence of resistant isolates in the goat kids remained high but decreased post weaning, reaching significantly lower levels by one year of age. Our findings are similar to those reported by many other authors [9,35,37,38,39] who detected higher colonization by resistant *E. coli* in young calves and pigs that declined with age of the animals. Our findings also agree with *Berge* et al 2010 finding in cattle who reported a higher prevalence of antimicrobial resistance in younger animals than older animals and also early colonization by resistant isolates in the absence of antibiotic use. We also found that while most of the isolates found in the young animals at 3 weeks of age were of intermediate resistance, this pattern of colonization changed over time and by the time the animals were three months and beyond, most resistant *E. coli* were of the full resistance phenotype. It is unclear why this pattern would occur although our hypothesis is animal (more mature immune system) in combination with bacterial factors (lower fitness of strains with intermediate resistance compared to other gut bacteria) may play a role in reduced survival of isolates with intermediate resistance in older hosts. To our knowledge, this phenomenon has not been studied or reported in other studies. We also found that the resistant phenotypes found in goat kids were unrelated to those detected in the nursing does at all sampling points during the pre-weaning period despite sharing the same ecosystem. Furthermore, resistant phenotypes detected in individual animals during the same period varied between sampling time points. This pattern may imply that acquisition and colonization by resistant *E. coli* isolates was an independent dynamic event and persistence in individual animals did not occur under the study conditions.

Antibiotic resistance to a broad range and groups of antibiotics were detected in *E. coli* in goats involved in this study either singly or in combination. Detected resistance included to tetracycline, streptomycin, ampicillin, amoxicillin/clavulanic acid, nalidixic acid, tobramycin, gentamicin, and chloramphenicol. As reported in other studies in food animals, most resistance was against tetracycline, streptomycin, ampicillin, and amoxicillin/clavulanic acid, while resistance to other antibiotics was low [40]. Resistance to tetracycline and streptomycin are the most commonly detected antibiotics in food animals, which is as expected because they have been in the market and use in food animals for a long time. In the current study, there was a history of previous use of tetracycline for treatment on the farm albeit over a year before the study commenced. It is possible that bacteria isolates harboring tetracycline resistance existed or persisted in the soil or in the gut of some older animals and colonized the study animals during grazing. This is supported by studies that have shown that once bacteria develop resistance to antibiotics, this phenotype can be detected as long as four years after cessation of the antibiotic use [34,41]. Persistence of tetracycline genes for several months in the soil amended or in contact with animal manure has also been reported in some studies [11,42]. In this study, some older animals that had been on site for over one year harbored tetracycline resistant isolates. Resistance to β-lactams, especially ampicillin, was detected in a significant number of *E. coli* isolates in this study despite no previous use of this antibiotic in the farm. This indicates that this kind of resistance may have been acquired from other environmental sources unrelated to activities on the farm. Our finding are similar to other reported results of resistance to ampicillin and other antibiotics reported in wild animals unrelated to known exposure to these antibiotics [4].

Differences in proportion and phenotype of antibiotic resistant *E. coli* was reported depending on whether goats were located on pasture or on smaller paddocks near holding facility. Significantly higher proportions of antibiotic resistant *E. coli* was detected in animals located at or near the housing facility than at pasture. This resistance was mostly due to *E. coli* resistant to tetracycline which was also more frequently detected in fecal samples from animals grazing near or at the housing facility than animals on pasture. Although not tested, it is possible that there was a higher level of antibiotic resistant bacteria in the immediate environment (soil) near the housing facility as a result of frequent congregation of animals during treatment resulting in a high load of resistant microbes in the soil. Studies have shown that soils exposed to manure with antibiotic resistant bacteria are persistent sources of antibiotic resistant bacteria [42]. On the other hand, *E. coli* isolates resistant to ampicillin and other antibiotics were more frequently isolated from animals on pasture than those near the facility pointing to a distant environmental source than on farm use. The location of the pasture is frequently visited by migratory birds due to the presence of fish ponds but also deer from adjacent woodlands. Additionally, there are human settlements surrounding the research farm. Thus, it may be that resistance to these antibiotics may have been spread to the farm by these wild animals from other places or surface water run-off from distant places [43,44]. In a previous study involving small ruminants from the current study farm and wild birds captured in the farm vicinity, *Salmonella* and *Campylobacter* isolates were found to have antimicrobial resistance to a wide range of antibiotics [45].

Most of the isolates in this cohort were resistant to either one or two antibiotics, while a few isolates had resistance to three antibiotics and one isolate to four antibiotics. This is a significant finding, given the fact there was no use of antibiotics in the cohort throughout the study period and these are animals predominantly reared on pasture. Resistant isolates found in these animals may reach far sites through surface water and runoff. Studies in poultry, swine, and cattle also report a high percentage of isolates with resistance to more than one antibiotic (reviewed in Reference [18]) in the US too. However, these animals are reared under intensive production systems in the US and in the past have been associated with relatively higher levels of use of antibiotics. 

The antibiotic resistance genes detected in the *E. coli* isolates in this study are similar to those found in other studies. Most of the antibiotic resistant genes detected in the study are located in plasmids and transposons that can be transferred between bacteria indicating the potential of goats as reservoirs of antibiotic resistant genes in the environment. The finding could also offer insight on the potential source of the resistant bacteria in absence of antibiotic use in these animals; an environmental pool of resistant genes in plasmids. In particular, *tetB* gene, which codes for a tetracycline efflux protein [46] was the only gene detected in tetracycline resistant isolates in this study. *TetB* was also the most frequent gene detected in *E. coli* isolates from pigs, chicken, and turkey although *tetA* was also detected [16,17,47]. Similarly, in a study involving *E. coli* isolates from companion animals, *tetB* gene was the most frequently detected [48]. Resistance to ampicillin in this study, was found to be mainly due to the *bla_-TEM_* gene which codes for beta-lactamases. The significance of this form of resistant mechanism is the fact that b*la_-TEM_* genes are located in transposons found in plasmids which play a key role in their dispersal among bacteria [49]. Our findings are in agreement with other studies involving *E. coli* isolates from foods of animal origin, healthy animals, and human that found that this gene was the most frequently detected in over 80% of ampicillin resistant *E. coli* [50], a Danish study in food animals found the same gene in 91% of ampicillin resistant isolates [51] and a study in Portugal involving companion animals detected the gene in 70% of ampicillin resistant isolates [48]. In contrast Srinivasan reported a high frequency of the inducible Amp C (91%) gene in *E. coli* isolates from mastitic cows that were resistant to ampicillin, indicating other possible mechanisms of ampicillin resistance in *E. coli*. In isolates resistant to streptomycin, the adenyl transferase mechanism of aminoglycoside inactivation by the *aadA* gene product was the most frequently detected in this study. The *strepA/strepB* gene was also detected in a few streptomycin resistant isolates. Similar to our findings, *aadA* gene was also detected in streptomycin *E. coli* isolates in companion animals [48] and also human and food animals [3]. Additionally, Srinivasan et al [52] reported high frequency for *aadA* (51%) gene and *strepA/strepB* genes (21.6%) in *E. coli* isolates from cows with mastitis. Isolates with tobramycin resistance had the aminoglycoside modifying enzyme (acetyl transferase) *aac(3)IV* gene detected which is known to responsible for resistance to tobramycin and amikacin [43] and was also reported in apramycin resistant *E. coli* from farm workers and farm animals [53]. The Serine 83 leucine and aspartate 87 asparagine mutations detected in nalidixic acid resistant isolates were similar to those reported in isolates from dogs in previous studies [48] and also retail meats in the US [54].

The concurrent detection of virulent genes in isolates that were also resistant to antibiotics was another significant finding in this study as they may be potential animal or public health pathogens. While *E. coli* isolates possessing the *stx1* and *hly*, *eae*, and *hly* virulent genes have been found in both healthy and diarrheic small ruminants, the full significance of these isolates has not been elucidated [53,55]. However, isolates possessing the *eae* and/or *stx2* genes are known to be pathogenic in both animals and human. In this study, most resistant isolates belonged to the phylogenetic group D followed by group B1. However, we also detected resistant *E. coli* isolates that belonged to the B2 phylogenetic group in which most virulent extra-intestinal strains are known to belong. Those belonging to phylogenetic group B1 and D may also be potential intestinal pathogens further underscoring the significance of the resistant *E. coli* isolates detected in this study. Contrary to our study, Camilla et al 2010 [56] did not find any *E. coli* strains belonging to phylogenetic D or B2 in *E. coli* isolates from goats in their study. This may be due to the small number of isolates analyzed in Reference (16) compared to our study (104). To our knowledge, this is the first study reporting on the temporal dynamics, shedding patterns, and significance of antimicrobial resistant *E. coli* from pastured goats.

## 5. Conclusions

The study findings indicate that pastured goats, despite low exposure to antibiotics, are colonized by antibiotic resistant bacteria early in life. Additionally, it highlights shedding dynamics with higher proportions of fecal shedding of antibiotic resistant *E. coli* by younger goat kids compared to older animals. Higher proportions of resistant isolates were also detected in animals congregated in paddocks that have been under intensive animal use/treatment for a long time compared to animals spread out on pasture. The study also found colonization by bacteria resistant to antibiotics that had never been used on the farm, indicating a source from a broader environmental resistance gene pool. The genes detected in resistant isolates were similar to genes detected in other food and companion animals and human resistant *E. coli* isolates. A significant number of resistant *E. coli* isolates from the goats also belonged to phylogenetic groups of public health importance and harbored virulent genes further emphasizing the significance of the isolates. Findings underscore the fact that small ruminants are reservoirs of antibiotic resistant *E. coli* that are also potentially pathogenic and could spread antibiotic resistant genes to other gut pathogenic or commensal bacteria and also to the environment.

## Figures and Tables

**Table 1:** Number and percentages of antibiotic resistance phenotypes in 136 resistant *E. coli* isolates from pastured meat goats.

Resistance Phenotype	Number of Isolates (*n* = 136)
Tetracycline(Tet)	50
Streptomycin(Strept)	25
Ampicillin(Amp)	17
Nalidixic acid(Nal)	6
Chloramphenicol (Chl)	2
Amikacin(Ak)	2
Amoxycillin/clavulanate(Amc)	1
Tobramycin(Tob)	1
Gentamicin(GN)	1
Sulfamethoxazole-trimethoprim(Sxt)	0
Meropenem	0
Ciprofloxacin	0
Tet/Strept	12
Amp/Amc	3
Tet/Amp	1
Tet/Nal	2
Tet/Chl	1
Amp/Chl	2
Tet/Amc	1
Tob/Strept	1
Chl/Tob	1
Amp/Nal	1
Ak/Tet	1
Amp/strept	1
Amc/Ak/Nal	1
Nal/Strept/Tet	1
Amp/Nal/Sxt	1
Amc/Chl/Tet/Strept	1
No resistance	272

**Table 2:** Percentage of *E. coli* isolates from pastured meat goats showing each individual antibiotic resistance.

Antimicrobial Agent	*n* = 136
Ampicillin	26
Streptomycin	41
Gentamicin	1
Tetracycline	70
Amikacin	4
Ciprofloxacin	0
Amoxycillin/Clavulanate	7
Meropenem	0
Choramphenicol	7
Tobramycin	3
Sulfamethoxazole-trimethoprim	1
Nalidixic acid	12

**Table 3:** Animal groups sampled and resistance phenotypes detected in 408 *E. coli* isolates from pastured meat goats.

Sampling Group	Isolate Phenotype			Number of Antibiotics Resistant to:		
	Sensitive	Resistant	Total	1	2	>2
Kids 3wks (P)	14	11	25	10	1	0
Kids 7wks (P)	13	11	24	11	0	0
Kids 11wks (F)	14	13	27	11	2	0
kids 13wks (F)	33	25	58	24	1	0
Kids 14wks (F)	11	11	22	9	2	0
Kids 6months (F/P)	32	22	54	14	7	1
Kids 1 year (P)	38	5	43	3	1	1
Nursing does (3wks) (P)	16	2	18	2	0	0
Nursing does (7wks) (P)	15	5	20	5	0	0
Nursing does (11wks) (F)	10	2	12	1	1	0
Nursing does (13wks) (F)	11	1	12	1	0	0
Adult does ** (P)	32	12	44	8	4	0
Other goats * (F)	33	16	49	6	8	2
Total	272	136	408	105	27	4

* 10-month goats in the same flock sampled near facility 2 months before the initial cohort was sampled at 3 weeks. ** Includes does that were in the initial cohort and * other goats sampled at pasture. F = facility; P = Pasture

**Table 4:** Resistance phenotype and the respective percentages of *E. coli* isolates detected in pastured goats at different locations at the research farm.

Antimicrobial Agent	Animal Location			
	Near facility (*n* = 212)	%	Pasture (*n* = 196)	%
Ampicillin	4*	2%	22**	11%
Streptomycin	18	9%	23	12%
Gentamicin	1	0.5%	0	0%
Tetracycline	65*	31%	5**	2.5%
Amikacin	4	2%	0	0%
Ciprofloxacin	0	0%	0	0%
Amoxycillin/Clavulate	4	2%	3	1.5%
Meropenem	0	0%	0	0%
Choramphenical	3	1.4%	4	2%
Tobramycin	1	0.5%	2	1%
Sulfamethoxazole-trimethoprim	0	0%	1	0.5%
Nalidixic acid	8	4%	4	2%
Total resistant isolates	85**	40%	51*	26%

* Values on the same row with different number of asterisks ** vs. * are significantly different *P* < 0.05.

**Table 5:** Resistance genes detected among antibiotic resistant *E. coli* isolates of pastured goats origin.

Phenotype of Resistance	Number of Isolates with This Phenotype	Gene Detected	Number of Isolates
Ampicillin	26	*blaTEM*	12
Amoxycillin/clavulanate	7	*blaSHV*	1
Tetracycline	70	*tet(B)*	69
Streptomycin	41	*aadA* *strB/strA*	18 10
Sulfamethoxazole-trimethoprim	1	*sul2*	1
Chloramphenicol	7	-	-
Tobramycin	3	*aac (3)/aac (3*)	1
Amikacin	4	-	-
Gentamicin	1	-	-

## Appendix A

**Table A1:** Primers used in study.

Primer Name	Sequence (5’ 3’)	Target Gene(s) or Region	Amplicon Size	Ref
TEM-F	TTCTTGAAGACGAAAGGGC	*bla_TEM_*	1150	[48]
TEM-R	ACGCTCAGTGGAACGAAAAC			
SHV-SE	ACTCAAGGATGTATTGTG	*blaSHV*	747	[57]
SHV-AS	TTAGGGTTGCCAGTGCTCG			
TEM-C	ATCAGCAATAAACCAGC	*bla_TEM_*	516	[58]
TEM-H	CCCCGAAGAACGTTTTC			
HE605	TTTCGTGTCGCCCTTATTCC	*bla_TEM_*	690	[59]
HE606	CCGGCTCCAGATTTATCAGC			
TEM-164.SE	TCGCCGCATACACTATTCTCAGAATGA	*bla_TEM_*	447	[57]
TEM-165.AS	ACGCTCACCGGCTCCAGATTTAT			
Tet A-F	GTAATTCTGAGCACTGTCGC	*tetA*	937	[48]
Tet A-R	CTGTCCTGGACAACATTGCTT			
Tet B-F	CTCAGTATTCCAAGCCTTTG	*tetB*	416	[48]
Tet B-R	CTAAGCACTTGTCTCCTGTT			
Tet C-F	TCTAACAATGCGCTCATCGT	*tetC*	570	[48]
Tet C-R	GGTTGAAGGCTCTCAAGGGC			
Tet D-F	ATTACACTGCTGGACGCGAT	*tetD*	1104	[48]
Tet D-R	CTGATCAGCAGACAGATTGC			
Tet E-F	GTGATGATGGCACTGGTCAT	*tetE*	1,179	[48]
Tet E-R	CTCTGCTGTACATCGCTCTT			
Str A-R	ATGGTGGACCCTAAAACTCT	*streptA/B*	893	[13]
Str B-F	CGTCTAGGATCGAGACAAAG			
strA-F	CTTGGTGATAAGGCAATTC	*strept A*	548	[40]
strA-R	CCAATCGCAGATAGAAGGC			
strA-F	GTCAAGGGATTGAAACC	*streptA/B*	509	[40]
strB-R	GGATCGTAGAACATATTGGC			
AadA F	GTGGATGGCGGCCTGAAGCC	*aadA*	525	[40]
AadA R	AATGCCCAGTCGGCAGCG			
Sul1-F	TGGTGACGGTGTTCGGCATTC	*sul1*	789	[48]
Sul1-R	GCGAGGGTTTCCGAGAAGGCC			
Sul2-F	CGGCATCAACATAACC	*sul2*	722	[48]
Sul2-R	GTGTGCGGATGAAGTCAG			
Sul3-F	GAGCAAGATTTTTGGAATCG	*sul3*	792	[48]
Sul3-R.	CATCTGCAGCTAACCTAGGGCTTTGGA			
GyrA-F	TACACCGGTCAACATTGAGG	*gyr*	648	[48]
Gyra-R.	TTAATGATTGCCGCCGTCGG			
CmlA.F	TGTCATTTACGGCATACTCG	*cml*	455	[60]
CmlA.R	ATCAGGCATCCCATTCCCAT			
aac(3)IV F	TGCTGGTCCACAGCTCCTTC	aac(3)IV	653	[61]
aac(3)IVR	CGGATGCAGGAAGATCAA			
Stx1-a	TCTCAGTGGGCGTTCTTATG	*stx1*	338	[62]
Stx1-b	TACCCCCTCAACTGCTAATA			
Stx2-a	GCGGTTTTATTTGCATTAGC	*stx2*	115	[62]
Stx2-b	TCCCGTCAACCTTCACTGTA			
EAE-a	ATGCTTAGTGCTGGTTTAGG	*eae*	248	[62]
EAE-b	GCCTTCATCATTTCGCTTTC			
HlyA-a	AGCTGCAAGTGCGGGTCTG	*hly*	569	[62]
HlyA-b	TACGGGTTATGCCTGCAAGTTCAC			
ChuA.1	GACGAACCA ACGGTCAGGAT	*chuA*	279	[33]
ChuA.2	TGCCGCCAGTACC AAAGACA			
YjaA.1	TGAAGTGTCAGGAGACGCTG	*yjaA*	211	[33]
YjaA.2	ATGGAGAATGCGTTCCTCAAC			
TspE4C2.1	GAGTAATGTCGGGGCATTCA	*tspE4.C2*	152	[33]
TspE4C2.2	CGCGCCAACAAAGTATTACG			
EC16S-a	CCCCCTGGACGAAGACTGAC	*E. coli* 16s	401	[62]
EC16S-b	ACCGCTGGCAACAAAGGATA			

## Appendix B

**Table A2:** Statistical results of comparisons of proportions of antibiotic resistant isolates analyzed using MedCalc Software.

Comparison Groups	Chi Square Value	*P* Value
Kids vs does 3 weeks	5.282	*P* = 0.0215
Kids vs does 7 weeks	2.03	*P* = 0.1543
Kids vs does 11 weeks	3.287	*P* = 0.0698
Kids vs does 13 weeks	5.151	*P* = 0.0232
Kids 7 weeks vs kids six months	2.611	*P* = 0.1061
Kids 7 weeks vs kids one year	9.734	*P* = 0.0018
Goats on pasture vs facility	8.968	*P* = 0.0027
Tetracycline resistance on facility vs pasture	57.654	*P* < 0.0001
Ampicillin resistance on pasture vs facility	14.742	*P* = 0.0001
